# A novel technique for moderate to severe intrauterine adhesions: A historical cohort study

**DOI:** 10.1097/MD.0000000000030480

**Published:** 2022-09-09

**Authors:** Huadi Yang, Xuelu Jiang, Ting Chen, Zhitao Yao, Xuqun Xu, Li Wu, Xiaojing Zhu

**Affiliations:** a Department of Gynecology and Obstetrics, The First Affiliated Hospital of Zhejiang Chinese Medical University, Hangzhou, Zhejiang Province, China; b Department of Ultrasonography, The First Affiliated Hospital of Zhejiang Chinese Medical University, Hangzhou, Zhejiang Province, China; c Department of Center of Clinical Evaluation, The First Affiliated Hospital of Zhejiang Chinese Medical University, Hangzhou, Zhejiang Province, China; d The First Clinical Medical College, Zhejiang Chinese Medical University, Hangzhou, Zhejiang Province, China.

**Keywords:** hysteroscopy, intrauterine adhesion, novel technique

## Abstract

To evaluate the feasibility and efficiency of our novel technique, ultrasound guided hysteroscopic catheter dilation (US-HCD), for the treatment of moderate to severe intrauterine adhesion (IUA). A total of 126 patients diagnosed with IUA and met the enrollment criteria were admitted in this historical cohort study from June 1, 2016 to December 31, 2018. All patients were divided into 2 groups according to the surgical techniques used. Group A (n = 68) were treated with traditional hysteroscopic adhesiolysis with scissors (THA) and Group B (n = 58) were treated with US-HCD. Their data for the next 2 years following the initial surgery were analyzed. Safety and feasibility (operation time, surgical complications and the third-look hysteroscopic surgery rate), and post-operation efficacy (reduction of American Fertility Society [AFS] scores, pregnancy and live birth rates) were evaluated between groups. Between the groups, there was no statistically significant differences in basic preoperative information and AFS scores (*P* > .05). While there were significant differences in the operation time of the initial surgery (*P* < .05) and reduction of AFS scores (*P* < .05). No surgical complications were recorded and only 3 patients (5.2%) received a third-look hysteroscopy in Group B, while there were 6 cases of complications and 13 cases (19.1%) of third-look hysteroscopy in Group A, indicating significant differences between Groups (*P* < .05). Both groups exhibited comparable pregnancy rate, live birth rate and obstetric complications (*P* > .05). Our new technique is a safe, feasible and effective procedure for moderate to severe IUA patients, which can be mastered more quickly and easily by surgeons and applied in areas with less affluent economy and without hysteroscopic scissors, thus worthy of further study.

## 1. Introduction

Intrauterine adhesion (IUA), which is used interchangeably with the term “Asherman’s syndrome,” mostly occurs after trauma to the basalis layer of the endometrium with any surgery performed within the uterine cavity.^[1]^ It is reported approximately 93% of IUA was caused by curettage after miscarriage.^[2]^

In China, IUAs are very common because the abortion rate is as high as 29.^[3]^ With the change of China’s family planning policy, more and more women diagnosed with IUA are eager to have a second or third child. However, IUA leads to partial or complete obstruction of the uterine cavity and/or the cervical canal and results in infertility or miscarriage. As hysteroscopic techniques have developed, diagnosis and treatment outcomes have been improved dramatically.^[4]^

Hysteroscopic adhesiolysis surgery with scissors is now the treatment of choice for IUA because of its minimally invasive nature and direct vision.^[1]^ Few studies have evaluated the outcomes after hysteroscopic adhesiolysis. Overall,^[5]^ restoration of normal menstruation is observed in 75% to 100%. The pregnancy rate ranges between 25% and 76%, and the term delivery rate, in women who achieved pregnancy, between 25% and 79.7%. Surgical complications includes uterine perforations, moderate or severe fluid overload, heavy uterine bleeding, technique replacements (e.g., transfer to use a resectoscope). Despite these shortcomings, little has been reported in terms of novel treatment technique in the past couple of decades. A technique with less complications and good effect is urgently needed.

Our medical group consists of Dr XJ, XX, ZY, each with >10 years’ experience in surgery for IUA, and HY, responsible for medical records. In 2016, we invented a novel surgical technique named *ultrasound guided hysteroscopic catheter dilation (US-HCD*). 2016 to 2018 we have used US-HCD to treat moderate to severe IUA patients and achieved significant results. We conduct this historical cohort study with the aim of sharing our experiences and assessing its efficacy, feasibility, and safety, comparing with the traditional hysteroscopic adhesiolysis with scissors (THA).

## 2. Method

### 2.1. Patients

**Figure 1.**
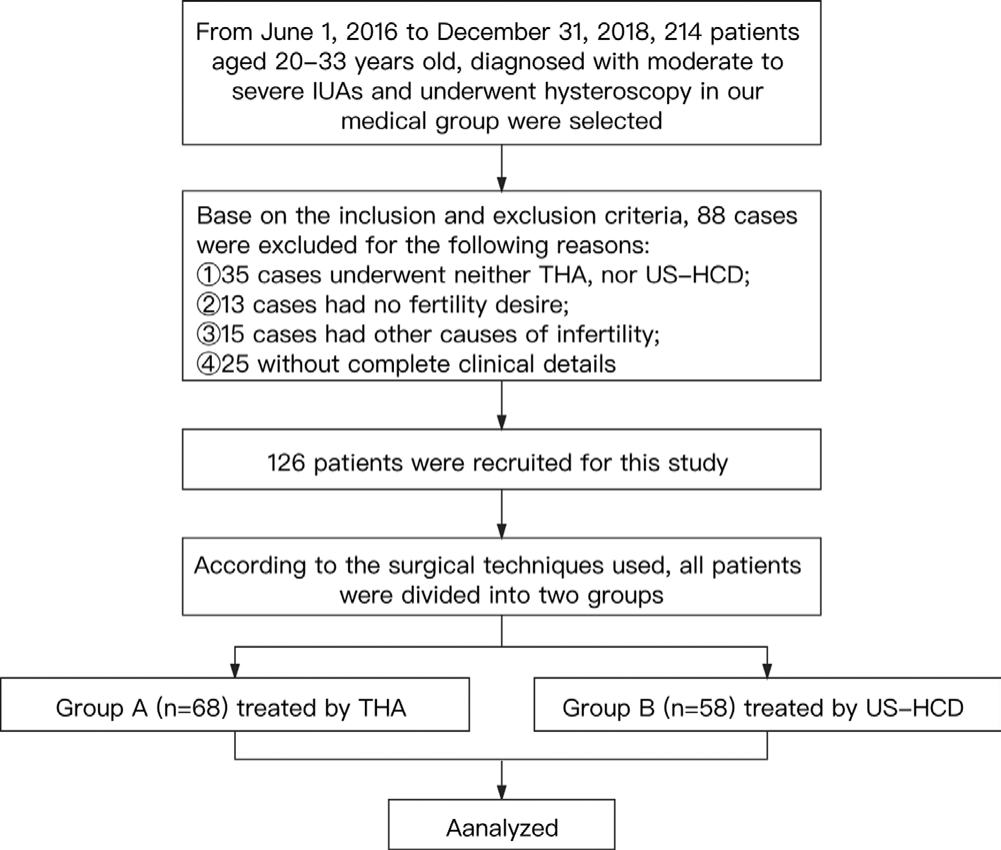
Selection of study participants.

The study began on January 1, 2020, and it was approved by the ethics committee of the first Affiliated Hospital of Zhejiang Chinese Medical University. We retrospectively reviewed the data of moderate to severe IUAs patients who got hysteroscopy in our medical group from June 1, 2016 to December 31, 2018. The patients, whose case data of 2 years following the surgery (including complete surgical records, fertility process, and obstetric and neonatal outcomes) were accessed in the database, were recruited for this study. The last patient recruited was observed until December 31, 2020. A flow chart explaining the patient selection process is presented in Figure 1.

The inclusion criteria based on the following criteria: undergoing hysteroscopic surgeries (either THA or US-HCD) by our surgical team first time at our hospital; moderate and severe IUAs confirmed by hysteroscopy according to the American Fertility Society (AFS) classification system^[6,7]^: 1 to 4 (mild), 5 to 8 (moderate), and 9 to 12 (severe); patients aged 20 to 33 years with fertility desire; normal endocrine function and ovulation. The exclusion criteria included: recurrent or tubercular IUA; other intrauterine diseases such as endometrial polyps or atypical hyperplasia; both tubal ostia were not exposed postoperatively or fallopian tubal blockage; grossly abnormal partner semen; severe systemic disease or contraindications to estrogen or surgery.

At last, 126 patients were included in this study. According to the surgical techniques used, all patients were divided into 2 groups, 68 patients who underwent THA were included in Group A and 58 patients who underwent US-HCD were included in Group B.

### 2.2. Surgical procedure

#### 2.2.1. Pre-operative general procedure.

US-HCD and THA were performed under intravenous anesthesia. All patients fasted for 6 to 8 hours before surgery. Phloroglucinol (40 mg) was intramuscular injected 20 minutes before surgery. A sterile saline solution was used to distend the uterus. Distension pressure was set to 110 to 120 mm Hg with a flow rate of 300 to 350 mL/minute.

#### 2.2.2. THA procedure.

1. Dilated the cervical canal to 7.5 mm.
2. Used a 6.5 mm operation hysteroscope to perform THA.
3. Confirmed the presence of IUA and evaluated the AFS prior to surgery to indicate the grade of adhesions.
4. Dissected the adhesions located in the central part of the uterine cavity by a 7 Fr rigid single action scissors.
5. Dissected the striped lateral adhesions (but not deal with the scar tissue).
6. Surgery endpoint: the normal uterine cavity and the tubal ostia were retrieved.

#### 2.2.3. US-HCD procedure.

**Figure 2.**
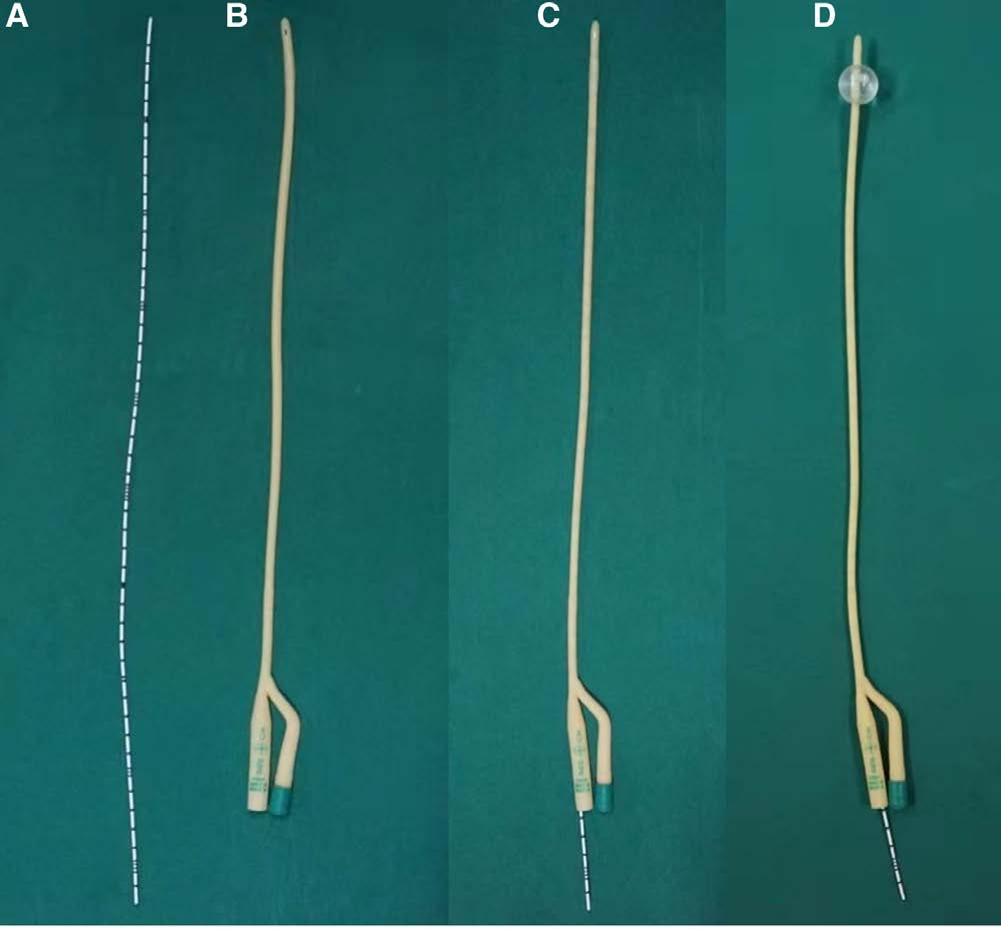
(A) A 14 Fr urethral catheter. (B) A F5 urethral catheter. (C) Our modified balloon catheter. (D) Modified balloon catheter with 6 to 8 mL of normal saline inside the balloon.

**Figure 3.**
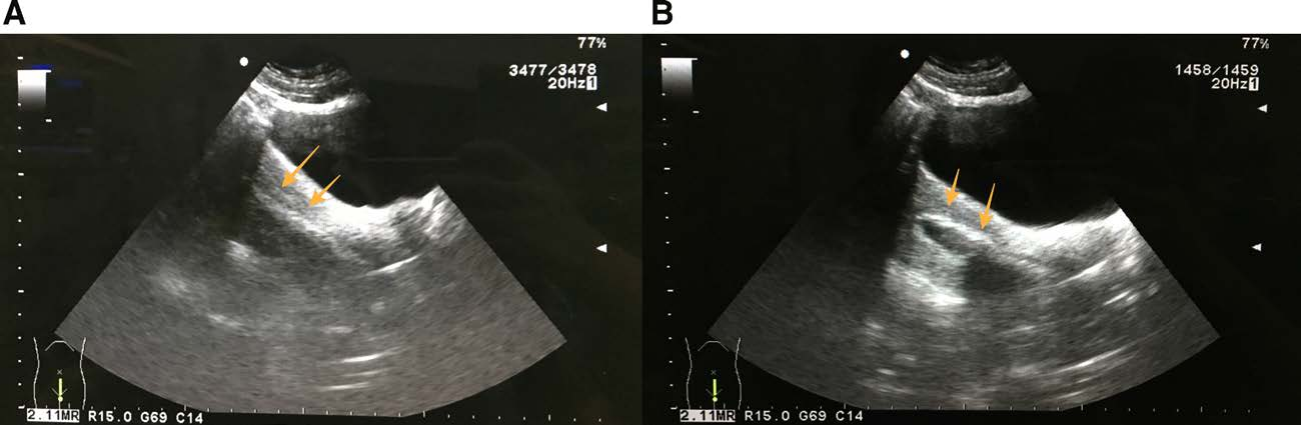
(A) US depicting modified balloon catheter with intrauterine placement. (B) US depicting catheter balloon dilation to resect adhesions. US = ultrasound.

**Figure 4.**
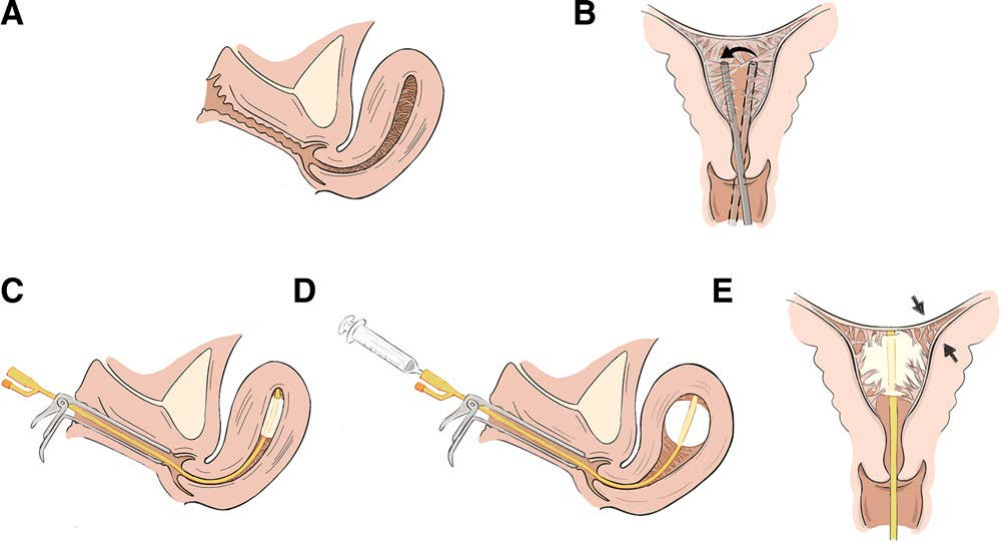
The process of US-HCD technique. (A) The existence of IUA. (B) Using the top of hysteroscopy to exclusively resect obvious central adhesions. (C) Placing the modified balloon catheter into the uterine cavity under US guidance. (D) Dilating the uterine cavity by slowly injecting normal saline into the balloon. (E) The scarred tissue on the walls and adhesions at the cornua uteri were both bluntly resected. IUA = intrauterine adhesion, US-HCD = ultrasound guided hysteroscopic catheter dilation.

**Figure 5.**
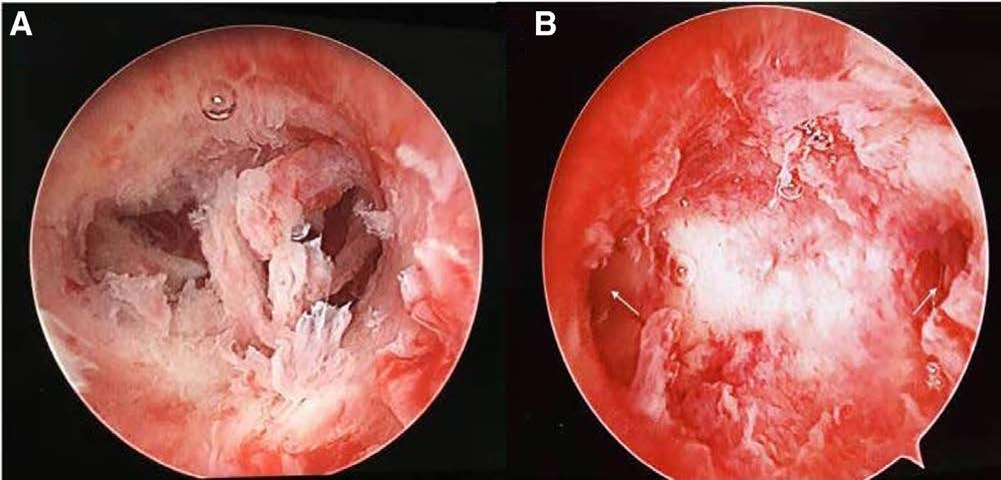
(A) Hysteroscopy revealing existence of adhesions and invisible cornua uteri. (B) Hysteroscopy revealing adhesion lysing and cornua uteri coming to light after surgery (the arrows pointed to).

1. The operation was monitored under transabdominal sonographic guidance (in order to easier visibility of the uterus on trans-abdominal US images, the patient’s bladder remained full or was back-filled).
2. Dilated the cervical canal to 7.5 mm.
3. Used a 6.5 mm operation hysteroscope to perform US-HCD, confirmed the presence of IUA and evaluated the AFS.
4. Used the top of hysteroscope to exclusively snap obvious central adhesions bluntly.
5. Placed a 14 Fr urethral catheter (Fig. 2A) inside a F5 bladder catheter (Fig. 2B) to make a modified balloon catheter (Fig. 2C and D). Then, placed the modified balloon catheter into the uterine cavity by US-guidance.
6. Dilated the uterine cavity by slowly injecting 6 to 8 mL of normal saline into the balloon to resect adhesions bluntly until there was no tense endometrial scar under US (Fig. 3).
7. Took out the balloon catheter and put the hysteroscope into the uterine cavity again. If obvious adhesion and sidewall scars were still present, the above procedure (Fig. 4) is repeated.
8. Surgery endpoint: besides the separated central adhesion, the scar tissue covering the walls of the uterine cavity was loosened, resulting in the exposure of the pink myometrium and the opened uterine cavity, the tubal ostia were visualized and the communication between the cavity and both the cervical canal and the fallopian tubes was facilitated as possible (Fig. 5).

#### 2.2.4. Postoperative general procedure.

Following surgery, a uterine-shaped stainless-steel intrauterine device (copper coil, Yandai Contraceptive Instrument Company, China) was inserted into the uterine cavity with its position checked via hysteroscopy to ensure that the size of the IUD matched the uterine cavity size and that the IUD was correctly positioned. Next, 3 mL hyaluronic acid gel (Changzhou Institute of Medicine, China)^[8]^ was placed inside the uterine cavity to reduce the recurrence of IUA.

#### 2.2.5. Postoperative management and observation.

The second-look hysteroscopic surgery was scheduled 8 weeks later to evaluate the initial surgical effect, and repeated hysteroscopic adhesiolysis that was the same as the initial surgery was performed if adhesion persisted until there was complete restoration of the endometrial cavity. A third-look hysteroscopy would be performed if there was still moderate to severe IUA confirmed by second-look hysteroscopy. The IUD was removed during the final hysteroscopy. The AFS scores were updated once again during the followed hysteroscopy. Patients were suggested to resume their conception efforts after surgery.

All patients’ data for 2 years following the initial surgery, including pregnancy and pregnancy outcomes, were analyzed.

### 2.3. Statistics

Statistical analysis was performed using SPSS® (v. 22.0; IBM Corp., New York, NY) for Windows. Continuous variables were presented as x ®±S. The AFS score average was reported using median and range. Changes of AFS score after therapy were compared using Wilcoxon signed rank tests within groups and using Wilcoxon Mann–Whitney tests between groups. Proportions between groups were compared by Chi-square tests, with Fisher exact test used as appropriate due to the small sample sizes. Comparisons pre- and post-treatment were undertaken using the Student *t* test. *P* < .05 was considered statistically significant.

## 3. Results

### 3.1. General conditions and clinical characteristics

The demographic data, presenting complaints, previous intrauterine operation, previous pregnancies history and grade of adhesions are documented in Table 1. No significant difference was observed between Groups for any of the variables assessed pre-operationally (*P* > .05) (Table 1). All women reported at least one pre-treatment symptom, including menstrual dysfunction, sub-fertility or pelvic pain, and intended to become pregnant post-treatment.

**Table 1:** Comparison of the baseline characteristics between 2 groups.

Group	Group A (n = 68)	Group B (n = 58)	*P* value
Characteristics ($\bar{x}\pm \text{s}$)			
Age (y)	28.03 ± 4.36	28.02 ± 3.74	.987*
Gravidity	2.01 ± 0.92	2.16 ± 0.99	.411*
Parity	0.74 ± 0.64	0.55 ± 0.50	.079*
**Presenting complaints, n (%)**			
Recurrent (≥2) miscarriage	8 (11.8)	2 (3.4)	.164†
Infertility	9 (13.2)	4 (6.9)	.383†
Amenorrhea	3 (4.4)	2 (3.4)	1.00†
Oligomenorrhea	41 (60.3)	44 (75.9)	.0673†
Cyclic pain	11 (16.2)	8 (13.8)	.709†
**Previous intrauterine operation, n (%)**			
D&C for induced abortion	34 (50.0)	34 (58.6)	.333†
D&C for delayed or incomplete miscarriage	16 (23.5)	15 (25.9)	.762†
Postpartum D&C	7 (10.3)	3 (5.2)	.466†
D&C for polyps or myomas	3 (4.4)	4 (6.9)	.828†
No intrauterine operation	4 (5.9)	2 (3.4)	.826†
**Previous pregnancies history, n (%)**			
Ectopic pregnancy	2 (2.9)	3 (5.2)	.856†
Caesarean delivery	12 (17.6)	10 (17.2)	.952†
Vaginal delivery	31 (45.6)	22 (37.9)	.385†
Adhesion grade, n (%)			
Moderate	60 (88.2)	52 (89.7)	.800†
Severe	8 (11.8)	6 (10.3)	.800†

D&C = dilation and curettage.

* Student *t* test.

† Chi-square test.

### 3.2. Operation time of the initial surgery

The operation time (the time from completed anesthesia to withdrawal of hysteroscopy) at the initial surgery of Group B was significantly reduced compared with that of Group A (*P* < .05) (Table 2).

**Table 2:** Comparison of clinical details between 2 groups.

Group	Group A (n = 68)	Group B (n = 58)	*P* value
Duration at the initial surgery (mm, $\bar{x}\pm \text{s}$)	23.4	20.3	<.000*
**Complications at initial surgery, n**	6	0	.030†
Uterine cavity perforation	1	0	-
False passage	3	0	-
Pelvic infection	2	0	-

* Student *t* test

† Fish test

### 3.3. Complications at surgery

In Group A, 6/68 occurred complications at surgery including uterine perforation, false passage and pelvic infection. Fortunately, the complications were controlled by drugs and no one exchanged to laparoscopic or transabdominal surgery. In Group B, no complications occurred (Table 2).

### 3.4. Comparison of AFS scores

AFS scores at each surgery in groups were compared in Table 3. Prior to surgery, there was no significant difference between the 2 groups (*P* = .392). At the second-look surgery, all the patients’ AFS scores reduced significantly compared with that of the first surgery (*P* < .000), and a significant difference in AFS scores was also noted between Groups A and B in second-look hysteroscopy merely (*P* < .05) (Table 3).

**Table 3:** Comparison of AFS scores and third-look hysteroscopy between 2 groups.

AFS scores (median [Q_L_~Q_U_])			
Median score prior to surgery	5 (5–10)	5 (5–9)	0.392*
Median score at second-look hysteroscopy	4 (0–7)	3 (0–7)	0.008*
P value	<.000†	<.000†	
Median Score at third-look hysteroscopy	3 (2–4)	4 (3–4)	0.117*
Third-look hysteroscopy, n (%)	13 (19.1)	3 (5.2)	0.038‡

* Wilcoxon Mann–Whitney tests.

† Wilcoxon signed rank test.

‡ Chi-square test.

### 3.5. Comparison of third-look surgery

Only 3 (5.2%) patients in Group B, while 13 (19.1%) cases in Group A, received a third-look hysteroscopy, and a significant difference was observed between Groups (*P* = .038) (Table 3).

### 3.6. Pregnancy outcome and obstetric outcomes

During a 24-month post-surgery period, 56 patients in Group A and 45 patients in Group B had one pregnancy. 98 of them conceived naturally, except for 3 patients in Group A received ART. And 32/53 and 25/45 had live birth without no multiple births. There is no significance difference between 2 groups in pregnancy outcome and obstetric outcomes (Tables 4 and 5).

**Table 4:** Pregnancy outcome between 2 groups.

Group	Group A (n = 68)	Group B (n = 58)	*P* value
**Pregnancy, n (%**)	56 (82.4)	45 (77.6)	.504†
** Spontaneous pregnancies**	53 (94.6)	45 (100)	.251†
Ongoing pregnancy	3 (5.7)	6 (13.3)	.297†
Miscarriages	15 (28.3)	11 (24.4)	.666†
Ectopic pregnancy	2 (3.8)	3 (6.7)	.851†
Live birth	32 (60.4)	25 (55.6)	.630†
Stillbirth	1 (1.9)	0 (0)	1.00*
Multiple gestation	0	0	–
** Assisted reproductive (ART), n (%**)	3 (5.4)	0 (0)	.251*
Ongoing pregnancy	3	0	–
Miscarriages	0	0	–
Ectopic pregnancy	0	0	–
Live birth	0	0	–
Stillbirth	0	0	–
Multiple gestations	1	0	–
** No pregnancy, n (%**)	12 (17.6)	13 (22.4)	.504†
Referred for ART	2	1	–
Treating with drugs	3	2	–
Treating with TCM	7	10	–

TCM = traditional Chinese medicine.

* Fish test

† Chi-square test.

**Table 5:** Obstetric outcomes from live births between 2 groups.

Group	Group A	Group B	*P* value
**Number of births, n (%**)	32	25	.056*
Caesarean delivery	15 (46.9)	9 (36.0)	.469*
Antepartum bleeding	2	1	-
Placenta previa	2	0	-
Placental abruption	1	1	-
Placenta acreta	1	0	-
Postpartum haemorrhage	3	0	-
Uterine rupture	0	0	-
Postpartum hysterectomy	0	0	-

* Chi-square test.

## 4. Discussion

Hysteroscopy have revolutionized treatment for IUA. Hysteroscopic adhesiolysis with scissors has since been the standard mode of therapy. However, it still presents a challenge. The more lateral the adhesions and the greater their density, the more difficult the dissection and the greater the risk of complications. Risks include perforation and creation of false passages, which may require patients to have additional surgical procedures. And it takes professional experience and a long operation time to deal with the adhesions at cornua uteri. And also, THA just separated the adhesion and leave the sidewall scarred tissue alone. While the contraction of the scarred tissue covering the uterine lateral wall is the main cause for the narrow uterine cavity, as it might block the blood supply to the endometrium and cause the uterine cavity to shrink. Treatment of these adhesions can maximize the recovery of the uterine cavity. Therefore, some surgeons prefer energy instruments, such as L-hook electrodes, to cut the sidewall scarred tissue. Though the scars were quickly dissected, the heat generated by electrical instruments may cause thermal injury to the endometrium.^[9]^ A safe and effective way is urgently needed. However, little has been reported in terms of novel treatment technique in the past couple of decades.

Any treatment of IUA has essentially 3 aims: to restore the size and shape of the uterine cavity, to restore normal endometrial function and to make pregnancy possible.^[10]^ To meet these aims, we invented US-HCD. In this study, US-HCD was compared with THA through retrospective analysis of their basic preoperation features and postoperation efficacy, feasibility, and safety. There were no statistical differences in terms of age, AFS scores, pregnancy history, uterine cavity operations in these 2 groups and the only difference between them was surgical approach. Finally, we come to the conclusion that US-HCD has the following advantages.

First, there was no complications. US-guidance ensures accurate access to the uterine cavity and avoid perforation and creation of false passages due to blind exploration. In US-HCD, we used the top of hysteroscope to exclusively snap obvious central adhesions bluntly and dilated the uterine cavity by balloon under US visualization, resulting in no perforation. While in THA, using scissors to resect dense marginal adhesions and cornua uteri adhesions carries an increased risk of perforation. And in US-HCD, shorter operation time may contribute to a smaller volume difference of the dilation medium, though there was no fluid overload in both groups.

Second, the AFS scores of US-HCD were significantly lower than those of THA postoperationly. Although both approaches do not release heat, avoiding thermal damage to the endometrium adjacent to the scars, another reason for the lower AFS for US-HCD was that bluntly dissection of adhesion by balloon dilation loosened the walls from the contraction of the scarred tissue and bluntly resected adhesions at the cornua uteri, opening the uterine cavity and facilitating communication between the cavity and both the cervical canal and the fallopian tubes. The endometrial tissue lying under or near the scars became exposed, and the endometrium had space to grow and repair. While in THA, it just separated the adhesions without dealing with scar tissue, which decreased revascularization and angiogenesis.

And for both approaches, we just dilated the cervical canal to 7.5 mm, which reduced mucosal damage caused by cervical dilatation and the incidence of postoperation cervical incompetence. Following surgery, IUD and 3 mL hyaluronic acid gel, as barriers,^[11]^ were placed inside the uterine cavity as the separation of 2 opposite sides with a rough surface, to prevent the contact of both sides, and subsequently decreased the risk of adhesion between the 2. A recent prospective randomized controlled trial^[12]^ found that the application of hyaluronic acid gel is beneficial for the outcome of pregnancy for women with IUA. Meta-analysis^[13,14]^ showed that compared with estrogen treatment after hysteroscopy, the use of hyaluronic acid gel reduced the mean adhesion score at the second hysteroscopy. And it is reported that better endometrial thickness values were observed in those who received hyaluronic acid gel combined with IUD.^[15]^

Both groups exhibited comparable pregnancy rate, live birth rate and obstetric complications. It may be due to our small number of observation cases and short observation time. Compared with THA, our approach is simple, low cost, and wide availability. Owing to the above advantages, it can be mastered more quickly and easily by surgeons and applied in somewhere with poor economy and without hysteroscopic scissors.

In conclusion, US-HCD followed by deposition of IUD and application of hyaluronic acid gel is a safe and effective procedure for moderate to severe IUA patients. In addition to the surgical procedure, many variables that may affect the outcome of pregnancy, including the frequency of sexual activity, economic conditions and so on, were not involved in this study. More well-designed and adequately powered randomized studies are needed to assess whether this approach affects the key reproductive outcomes in a target population of infertile women.

This study was supported by Natural Science Foundation of Zhejiang Province, China [NO. LQ20H270003], National Natural Science Foundation of China [NO. 82104909] and Zhejiang Traditional Chinese Medicine Administration [NO. 2022ZB128].

## Author contributions

**Conceptualization:** Huadi Yang, Xuelu Jiang, Ting Chen.

**Data curation:** Huadi Yang, Ting Chen, Zhitao Yao, Xuqun Xu.

**Formal analysis:** Huadi Yang, Xuelu Jiang, Li Wu.

**Funding acquisition:** Huadi Yang.

**Writing – review & editing:** Huadi Yang, Ting Chen.

**Writing – original draft:** Xiaojing Zhu.
